# Sub-therapeutic vasopressin but not therapeutic vasopressin improves gastrointestinal microcirculation in septic rats: A randomized, placebo-controlled, blinded trial

**DOI:** 10.1371/journal.pone.0257034

**Published:** 2021-09-23

**Authors:** Jan Schulz, Inge Bauer, Anna Herminghaus, Olaf Picker, Richard Truse, Christian Vollmer

**Affiliations:** Department of Anesthesiology, University Hospital Duesseldorf, Duesseldorf, North Rhine-Westphalia, Germany; University of Calabria, ITALY

## Abstract

**Introduction:**

Sepsis impairs gastrointestinal microcirculation and it is hypothesized that this might increase patient’s mortality. Sub-therapeutic vasopressin improves gastric microcirculation under physiologic conditions whereas a therapeutic dosing regimen seems to be rather detrimental. However, the effects of sub-therapeutic vasopressin on gastrointestinal microcirculation in sepsis are largely unknown. Therefore, we conducted this trial to investigate the effect of sub-therapeutic as well as therapeutic vasopressin on gastrointestinal microcirculation in sepsis.

**Methods:**

40 male Wistar rats were randomized into 4 groups. Colon ascendens stent peritonitis (CASP)-surgery was performed to establish mild or moderate sepsis. 24 hours after surgery, animals received either vasopressin with increasing dosages every 30 min (6.75, 13.5 (sub-therapeutic), 27 mU · kg^-1^ · h^-1^ (therapeutic)) or vehicle. Microcirculatory oxygenation (μHBO_2_) of the colon was recorded for 90 min using tissue reflectance spectrophotometry. Intestinal microcirculatory perfusion (total vessel density (TVD; mm/mm^2^) and perfused vessel density (PVD; mm/mm^2^)) were measured using incident dark field-Imaging at baseline and after 60 min.

**Results:**

In mild as well as in moderate septic animals with vehicle-infusion intestinal μHbO_2_, TVD and PVD remained constant. In contrast, in moderate sepsis, sub-therapeutic vasopressin with 13.5 mU · kg^-1^ · h^-1^ elevated intestinal μHBO_2_ (+ 6.1 ± 5.3%; p < 0.05 vs. baseline) and TVD (+ 5.2 ± 3.0 mm/mm^2^; p < 0.05 vs. baseline). μHBO_2_, TVD and PVD were significantly increased compared to moderate sepsis alone. However, therapeutic vasopressin did not change intestinal microcirculation. In mild septic animals sub-therapeutic as well as therapeutic vasopressin had no relevant effect on gastrointestinal microcirculation. Systemic blood pressure remained constant in all groups.

**Conclusion:**

Sub-therapeutic vasopressin improves gastrointestinal microcirculatory oxygenation in moderate sepsis without altering systemic blood pressure. This protective effect seems to be mediated by an enhanced microcirculatory perfusion and thereby increased oxygen supply. In contrast, therapeutic vasopressin did not show this beneficial effect.

## Introduction

The gastrointestinal microcirculation maintains, through sufficient oxygen and nutrient supply, an adequate mucosal cellular function [1]. Sepsis impairs gastrointestinal microcirculation and it is hypothesized that this might lead to mucosal barrier failure and subsequent translocation of bacteria and toxins into the bloodstream and local lymph system. This probably increased gut permeability seems to play a role in the development of sepsis and multi-organ failure [2,3]. Thus, in the therapy of critical illness there is still ongoing research to optimize gastrointestinal microcirculation [4–7].

In previous studies, we could show that hypercapnic ventilation ameliorates the impaired gastrointestinal microcirculation in hemorrhagic shock and sepsis and seems to restore gut permeability [8–11]. However, with additional vasopressin receptor blockade these effects were abolished, indicating an involvement of the endogenous vasopressin system [8,12]. It is well known, that hypercapnia induces a slight increase of the vasopressin plasma concentration under physiologic as well as under septic conditions [8,13,14], which might explain the observed effects.

Vasopressin is a peptide, which is crucial for osmoregulation, homeostasis and especially cardiovascular control [15]. Its potent vascular effects are supported by the fact that vasopressin deficiency can contribute to vasodilatory shock states, in particular during sepsis [16]. Thus, exogenous vasopressin seems to be a tempting therapy to restore vascular tone and blood pressure in sepsis. Consequently, vasopressin is recommended in septic shock refractory to catecholamine treatment [17]. However, over-the-top use of exogenous vasopressin is known to impair regional microcirculation due to excessive vasoconstriction, especially in the gut [18]. Westphal and colleagues demonstrated in septic rats that high, far beyond clinically used dosages of vasopressin (360 mU · kg^-1^ · h^-1^) deteriorate splanchnic microcirculation [19]. Furthermore, also therapeutic vasopressin-infusions (2–4 U · h^-1^) seem to induce gastrointestinal hypoperfusion [20,21]. In contrast, we observed that sub-therapeutic vasopressin ameliorated gastrointestinal microcirculation under physiologic conditions [22]. Thus, it seems to be important to elucidate, if sub-therapeutic vasopressin can also be beneficial under septic conditions.

Another question in the current discussion is which septic patient does eventually profit from supplementary vasopressin-infusions. A retrospective analysis of a randomized clinical trial revealed that vasopressin seems to be only beneficial in patients with less severe sepsis [23].

To address these uncertainties, we conducted a randomized, placebo-controlled, blinded trial in septic rats with an sub-therapeutic as well as therapeutic dosing regimen of continuously infused vasopressin to determine the effects on the gastrointestinal microcirculation. Furthermore, we evaluated two different sepsis manifestations (mild and moderate) to evaluate if severity of sepsis influences the vasopressin effect.

## Materials and methods

### Ethic approval and consent to participate

All parts of this study were performed in accordance with NIH guidelines for animal care and reported in accordance with the ARRIVE guidelines. Experiments started after the agreement of the local Animal Care and Use Committee (Landesamt für Natur, Umwelt und Verbraucherschutz, Recklinghausen, Germany, Az. 84–02.04.2012.A361 (Institutional Care and Use Committee (IACUC)).

### Surgical induction of sepsis

**Fig 1:**
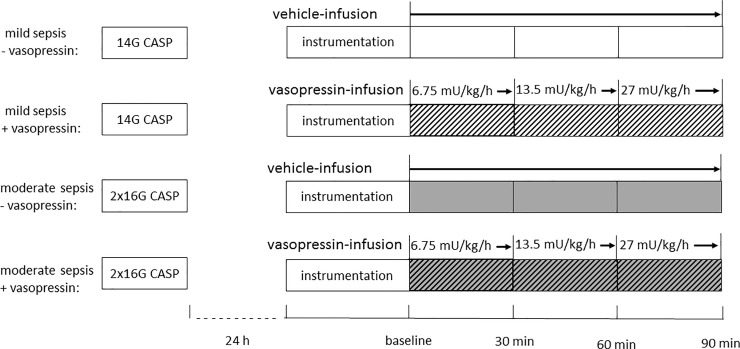
Experimental protocol. Colon ascendens stent peritonitis (CASP)-surgery was carried out 24 h before the experiment. Time controlled treatment with application of vehicle- or vasopressin-infusion. 4 different groups are formed: Mild sepsis—vasopressin: 14G-CASP operation with vehicle infusion, mild sepsis + vasopressin: 14G-CASP operation with dose escalating vasopressin-infusions, moderate sepsis—vasopressin: 2x16G-CASP operation with vehicle infusion, moderate sepsis + vasopressin: 2x16G-CASP operation with dose escalating vasopressin-infusions. Doses of 6.75 and 13.5 mU · kg^-1^ · h^-1^ represent sub-therapeutic vasopressin; 27 mU · kg^-1^ · h^-1^ is comparable to common therapeutic vasopressin dose.

48 male Wistar rats (320–380 g body weight) were randomly assigned to one of the 4 experimental groups (Fig 1). However, 8 animals died within 24 hours after induction of sepsis (3 mild septic animals and 5 moderate septic animals), so experiments were performed in 40 rats (n = 10) (Fig 1). It is of note, that there was no statistically relevant difference between the groups concerning death within the first 24 hours. After randomization the animals were derived from the breeding facility at the Central Animal Research Facility of the Heinrich-Heine-University Duesseldorf. The experiments started at 8:00 a.m. in the research laboratory of the Heinrich-Heine-University Duesseldorf, Dept. of Anesthesiology. In half of them colon ascendens stent peritonitis (CASP)-surgery was performed with a 14-gauge peripheral venous catheter (PVC) to establish a mild sepsis and in the other half with two 16-gauge PVC to develop a moderate sepsis manifestation using an established protocol as described previously [9,24]. Our previous studies demonstrate this model to be adequate to induce either mild or moderate sepsis in contrast to irreversible septic shock models. Anesthesia was induced and maintained in all animals by sevoflurane (3.0–3.2% end-expiratory, F_i_O_2_ 0.5) and buprenorphine (0.05 mg • kg^-1^, s.c.). A 2 cm-long median laparotomy was performed, the colon was located and penetrated 1 cm distal to the ileocecal valve, either with a 14-gauge PVC (Vasofix safety, B. Braun Melsungen AG, Melsungen, Germany) or with two 16-gauge PVC. The inner needles were withdrawn, allowing constant fecal leakage into the abdominal cavity to develop abdominal sepsis. The intestine was carefully returned and the abdominal wall was closed.

After CASP-surgery, animals were kept individually in separate plastic cages at a 12-h light/dark cycle with free access to water and food under controlled temperature (24 ± 2°C) and humidity (50% ± 5%). Buprenorphine (0.05 mg • kg^-1^ s.c.) was applied again 12 h after surgery. As described previously, animals were examined and scored every 6 h according to a defined protocol (Septic Rat Severity Score: SRSS-System) to determine the severity of sepsis and to monitor the animals with respect to their welfare (loss of body weight, appearance, spontaneous behavior, provoked behavior, breathing frequency, expiratory breathing sound, abdominal palpation and condition of droppings) [25,26]. Animals with unjustifiable suffering equivalent to a scoring of more than 10 points were euthanized. The scoring of all animals was performed by the same investigator.

### Assessment of the microcirculation

24 h after induction of sepsis, the animals were anesthetized by pentobarbital injection (60 mg • kg ^-1^ body weight i.p.), placed on a heating pad, tracheotomized and mechanically ventilated in a volume-controlled, pressure-limited mode (70 min ^-1^, VT 1.8–2.5 ml, PAW < 17 cm • H_2_O, PEEP 2 cm • H_2_O, FiO_2_ = 0.3, FiN_2_ = 0.7, Inspira Advanced safety Ventilator, Harvard Apparatus GmbH, March-Hugstetten, Germany). Throughout the experiment 120 μl blood were extracted intermittently for blood gas analysis (BGA) (ABL 715, Radiometer, Copenhagen, Denmark) to check for normocapnic ventilation (p_a_CO_2_ target value 38 ± 5 mmHg) and sufficient oxygenation (p_a_O_2_ target value 120–150 mmHg). If the target values were not achieved, ventilation or FiO_2_ were adjusted. To ensure continuous anesthesia, an external jugular vein catheter was established with subsequent continuous pentobarbital infusion (10 mg • kg^−1^ • h^−1^). Additionally, 2 mg pancuronium were injected. Blood pressure and heart rate were measured in the left *arteria carotis communis*, 1 ml arterial blood was extracted for Interleukin-6 (IL-6) measurements (see below). A continuous infusion of Ringer solution (4 ml • h^-1^) was applied via the arterial access for volume replacement and to prevent blood coagulation.

The animals were re-laparotomized and a flexible light guide probe (O_2_C LW 2222, Lea Medizintechnik GmbH, Gießen) was placed on the *tunica serosa* of the colon ascendens, 1 cm distal to the stent and also non-traumatic under the tongue of the rats (sublingual). Microcirculatory oxygenation (μHBO_2_) was measured as previously described via reflectance spectrophotometry [9]. In brief, this technique assesses microcirculatory oxygenation through the whole colonic wall (penetration depth of the probe: 0.7 mm; wall thickness 0.45 mm) and sublingual mucosa. White light (450–1000 nm) was transmitted to the colonic tissue via a micro-light guide and the reflected light is analyzed. The wavelength-dependent and overall absorption of the applied white light can be used to calculate the percentage of oxygenated hemoglobin in the microcirculation (μHbO_2_). Only the microcirculation is measured as light entering vessels bigger than 100 μm is completely absorbed. The biggest fraction of the blood volume is stored in venous vessels (85%), so predominantly postcapillary oxygenation is measured, which represents the critical partial pressure of oxygen (pO_2_) for hypoxia [27]. Online evaluation of the signal quality throughout the experiments allows verification of the correct position of the probe tip. The μHbO_2_ values reported are the means of the last 5 min every 30 min, beginning at baseline (Fig 1).

Furthermore, intestinal microcirculation was measured using incident dark field (IDF)—Imaging (Cytocam™, Braedius Medical, Huizen, The Netherlands) at baseline time point and after 60 min [28]. At every time point, three video sequences were recorded with 25 frames/s for 4 s at different cecal spots. Cytocam-IDF can be regarded as third-generation handheld microscope that allows real-time *in vivo* visualization of the microcirculation, as previously reported [29]. In brief, IDF Imaging uses high-brightness LEDs which emit light with a wavelength of 548 nm, to ensure the highest absorption of oxyhemoglobin and deoxyhemoglobin, and a short pulsed illumination time to visualize the red blood cells within the vessels. The technique can thereby distinguish between vessels which are perfused and vessels which are not perfused. The image acquisition is synchronized with a computer-controlled image sensor, so the image can be projected and also recorded on a computer. Following the data collection, each video sequence underwent a scan for exclusion quality criteria and was then analyzed with CytoCamTools 1.7.12 Software (Braedius Medical, Huizen, The Netherlands) to obtain microcirculatory parameters, according to the current international consensus for preclinical research: total vessel density (TVD; mm/mm^2^) and perfused vessel density (PVD; mm/mm^2^) [30–32]. The mean of the three measurements at each time point is reported.

After baseline measurements were completed, animals received either a vasopressin infusion (Arg-Vasopressin V9879 acetate salt, Sigma-Aldrich, Taufkirchen, Germany) or a vehicle infusion (NaCl) (Fig 1). Thereby, the investigator was blinded to the infusion contents. The animals receiving vasopressin were infused with increasing dosages in 30-min increments of vasopressin (6.75, 13.5, 27 mU · kg^-1^ · h^-1^). Thereby, concentrations of 6.75 and 13.5 mU · kg^-1^ · h^-1^ vasopressin are referred to as sub-therapeutic and clinically used vasopressin-infusion with 27 mU · kg^-1^ · h^-1^ is considered as therapeutic.

Thus, 4 different groups were analyzed: mild septic animals with vehicle-infusion (mild sepsis—vasopressin), mild septic animals with dose escalating vasopressin-infusions (mild sepsis + vasopressin), moderate septic animals with vehicle-infusion (moderate sepsis—vasopressin), moderate septic animals with dose escalating vasopressin-infusions (moderate sepsis + vasopressin) (Fig 1). At the end of the experiments, the animals were euthanized by exsanguination under deep anesthesia.

### IL-6 measurements

The blood sample was taken before initiation of baseline (see above). The blood was centrifuged (4°C, 4000 rpm, 10 min) and the plasma stored at –70°C for later analysis. IL-6 plasma levels were measured with an ELISA (BD Opt EIA^TM^, BD Biosciences, CA, USA) according to the manufacturer’s instructions.

### Statistical analysis

To calculate the appropriate sample size an *a priori* power analysis (G*Power Version 3.1.7, University of Dusseldorf, Germany) was performed. With n = 10 animals per group at a given α ≤ 0.05 (two-tailed) and an expected mean difference in μHbO_2_ of at least 20% (percentage points) with an expected standard deviation of 10–15% (based on previous studies) a power of 84.5% resulted.

Normal distribution of data was assessed and confirmed in Q-Q- plots (IBM SPSS Statistics, International Business Machine Corp., Armonk, New York, USA). Data were analyzed with a two-way ANOVA for repeated measures, followed by Dunnett´s post - hoc test for differences versus baseline, and Tukey post-hoc test for differences between groups. For IL-6 and SRSS data a one-way ANOVA was chosen, followed by a Tukey post-hoc test (GraphPad software v 6.0, Int., La Jolla, USA). Data are presented as means ± SD, p < 0.05 was considered significant.

Wherever delta values are presented, the absolute baseline value was subtracted from the absolute value at the respective observation points to individualize the data to each rat’s baseline.

## Results

**Fig 2:**
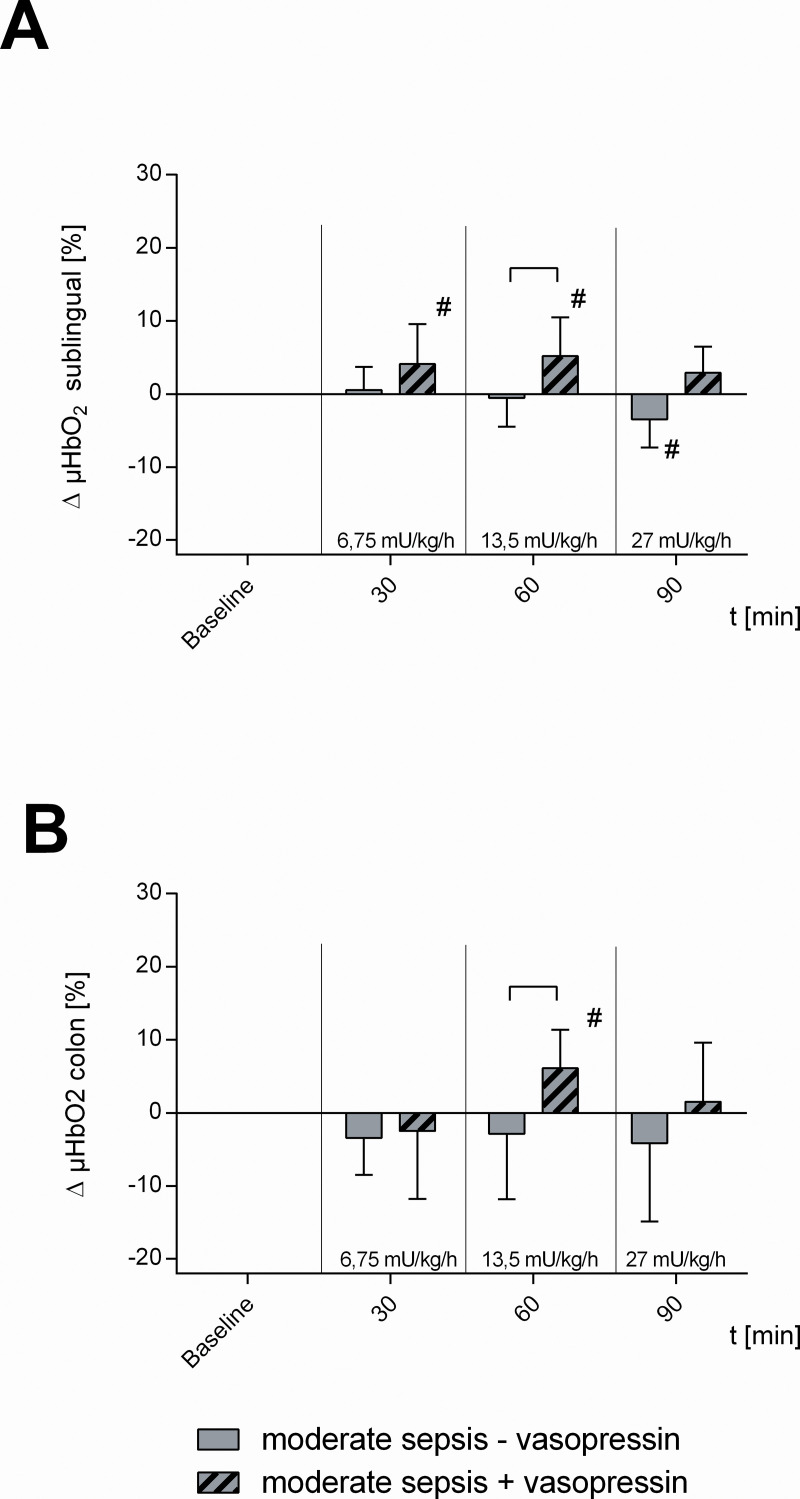
Microcirculatory oxygenation in moderate septic animals. Effect of vehicle- (moderate sepsis—vasopressin) and dose escalating vasopressin-infusions (moderate sepsis + vasopressin) on A) sublingual and B) colonic microcirculatory oxygenation (μHBO_2_) in moderate septic animals. Δ μHBO2 [%] of septic animals over time calculated to baseline (means ± SD). # = p < 0.05 versus baseline (Two-way ANOVA Dunnett) between groups (Two-way ANOVA Tukey); n = 10.

**Fig 3:**
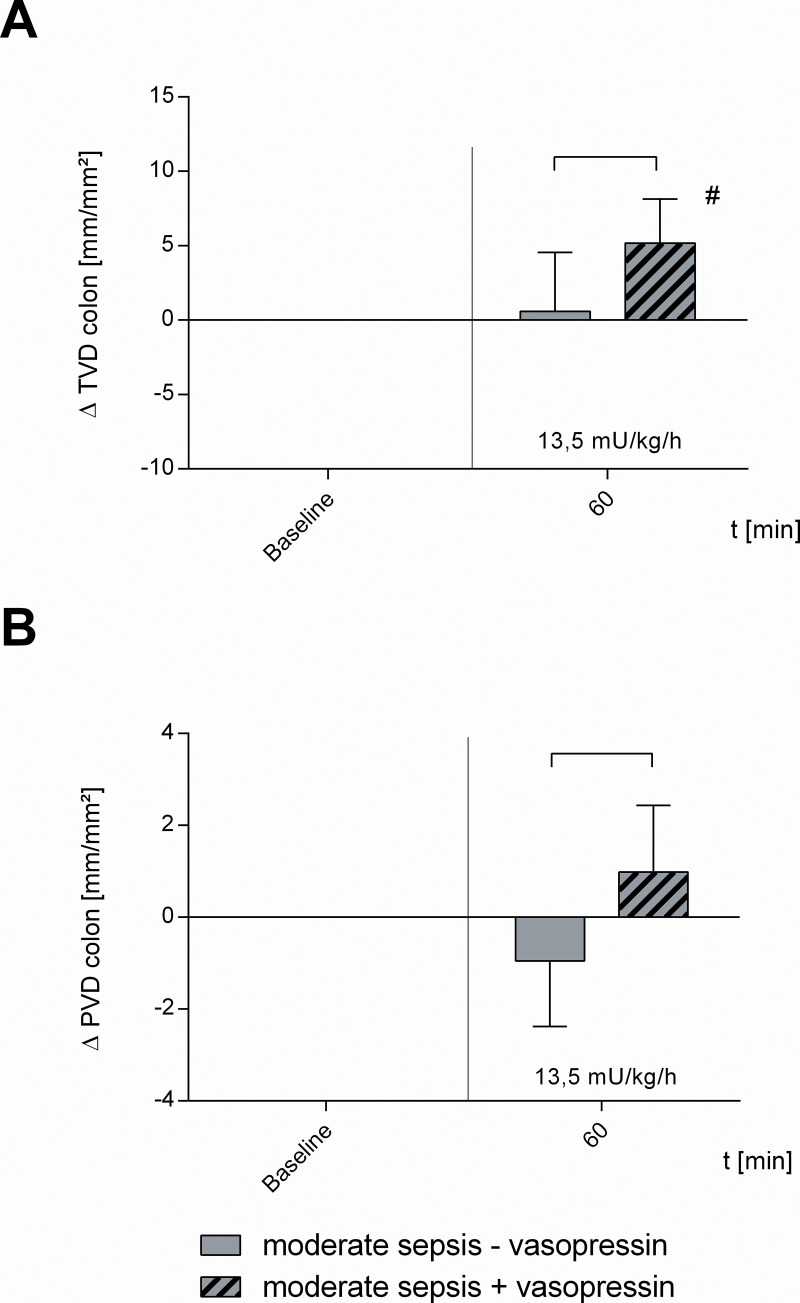
Microcirculatory perfusion in moderate septic animals. Effect of vehicle (moderate sepsis—vasopressin) and 13.5 mU · kg^-1^ · h^-1^ vasopressin (moderate sepsis + vasopressin) on A) colonic total vessel density (TVD) and B) perfused vessel density (PVD) in moderate septic animals. Δ TVD [mm/mm^2^] and Δ PVD [mm/mm^2^] of septic animals over time calculated to baseline (means ± SD); # = p < 0.05 versus baseline (Two-way ANOVA Dunnett) between groups (Two-way ANOVA Tukey); n = 10.

**Fig 4:**
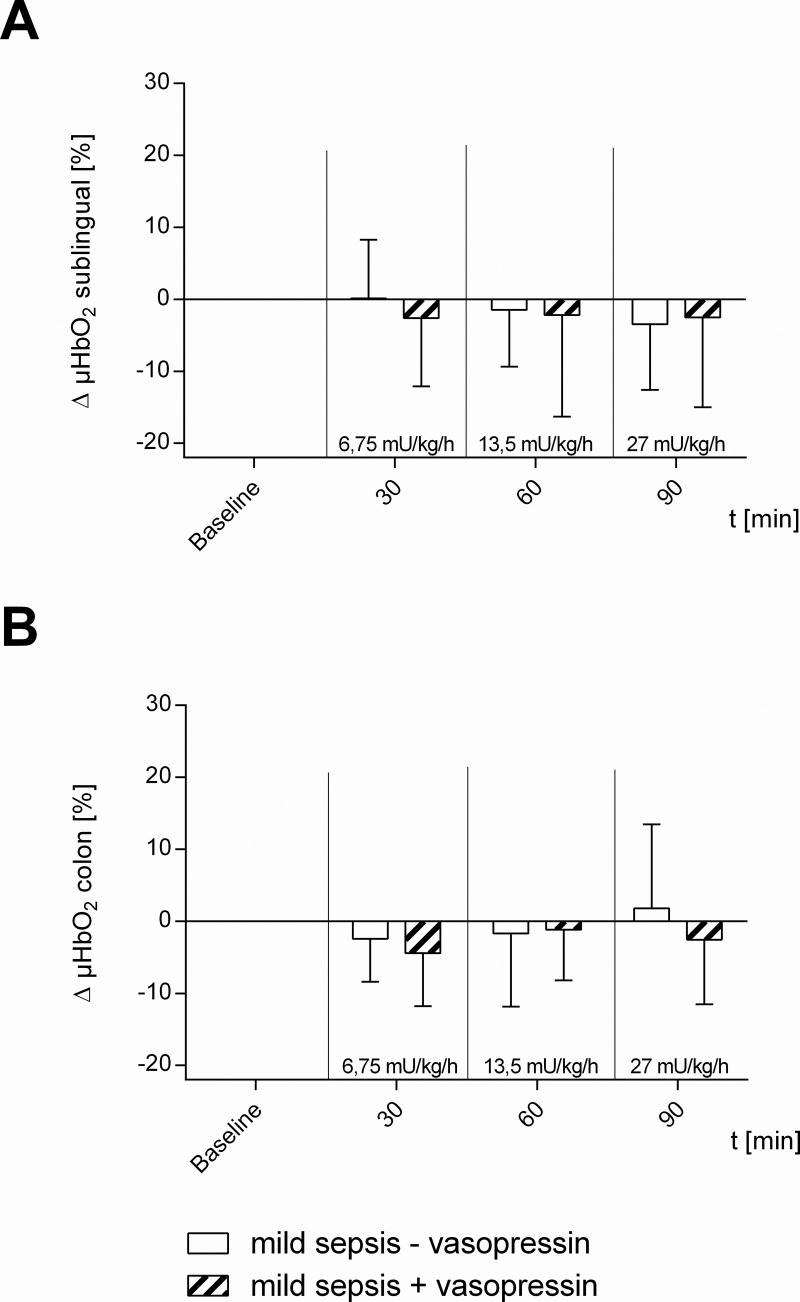
Microcirculatory oxygenation in mild septic animals. Effect of vehicle- (mild sepsis—vasopressin) and dose escalating vasopressin-infusions (mild sepsis + vasopressin) on A) sublingual and B) colonic microcirculatory oxygenation (μHBO_2_) in mild septic animals. Δ μHBO2 [%] of septic animals over time calculated to baseline (means ± SD); n = 10.

**Fig 5:**
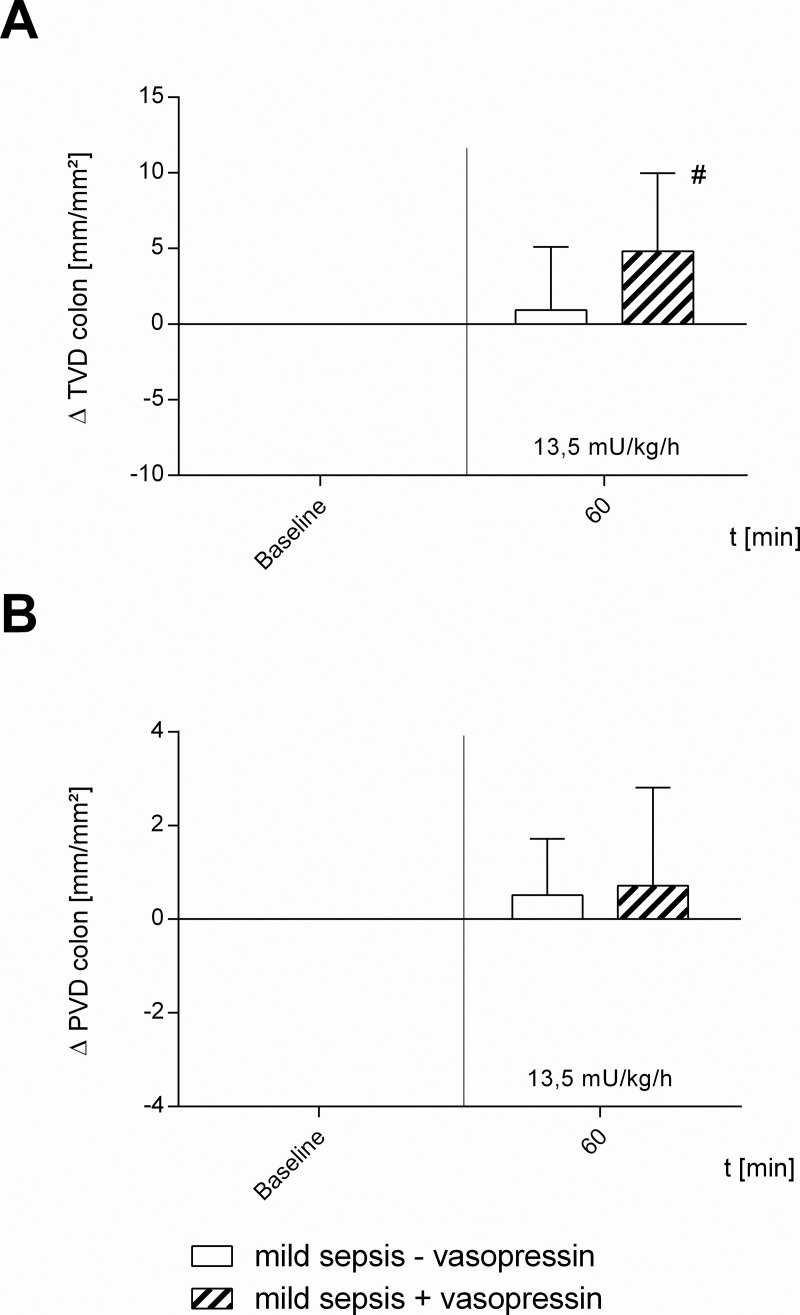
Microcirculatory perfusion in mild septic animals. Effect of vehicle (mild sepsis—vasopressin) and 13.5 mU · kg^-1^ · h^-1^ vasopressin (mild sepsis + vasopressin) on A) colonic total vessel density (TVD) and B) perfused vessel density (PVD) in mild septic animals. Δ TVD [mm/mm^2^] and Δ PVD [mm/mm^2^] of septic animals over time calculated to baseline (means ± SD); # = p < 0.05 versus baseline (Two-way ANOVA Dunnett); n = 10.

**Fig 6:**
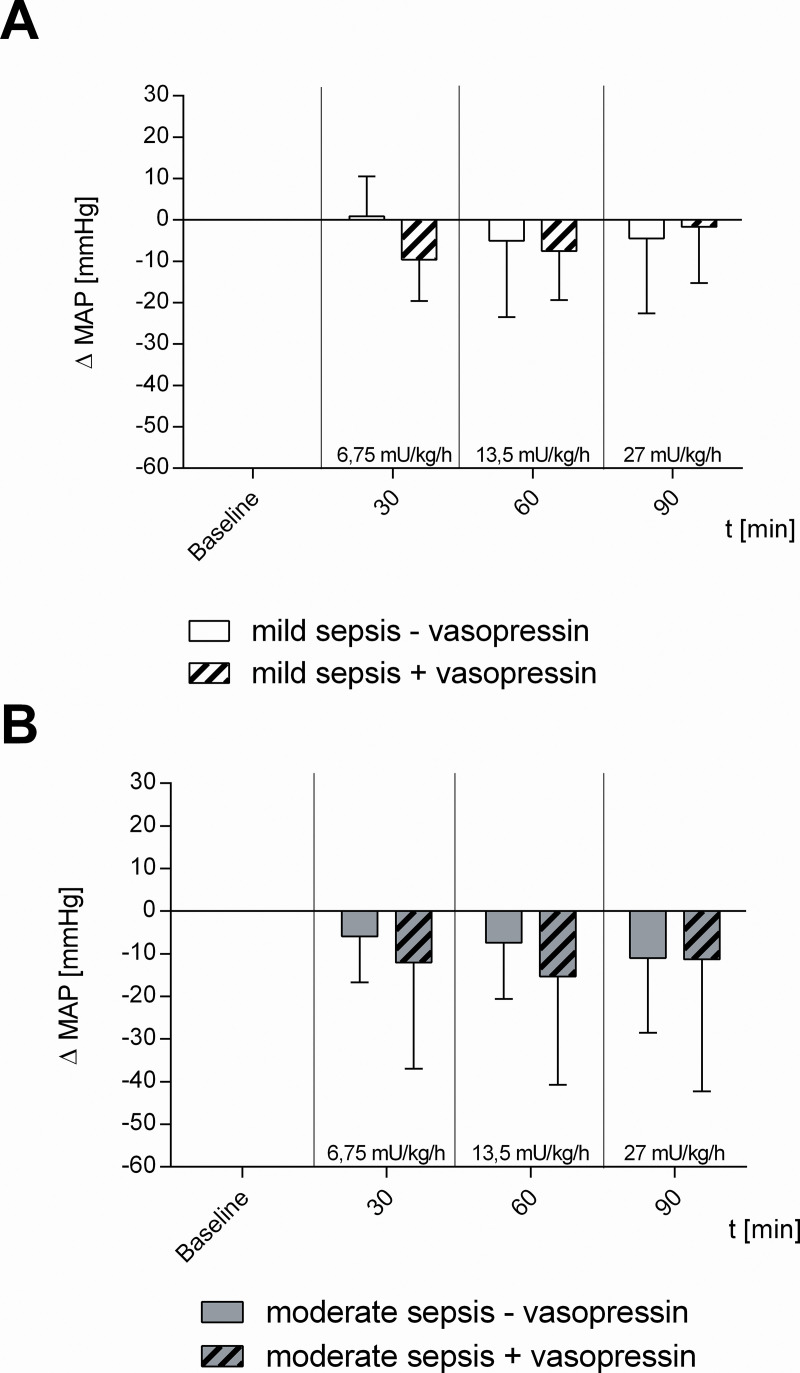
Mean arterial pressure. A) Effect of vehicle- (mild sepsis—vasopressin) and dose escalating vasopressin-infusions (mild sepsis + vasopressin) on mean arterial blood pressure (MAP) during mild sepsis. B) Effect of vehicle- (moderate sepsis—vasopressin) and dose escalating vasopressin-infusions (moderate sepsis + vasopressin) on MAP during moderate sepsis. Δ MAP [mmHg] of septic animals over time calculated to baseline (means ± SD); n = 10.

Table 1 and Figs 2–6 summarize the effects of vasopressin-infusion on systemic hemodynamics as well as sublingual and intestinal microcirculation in sepsis with different severity. 24 h after CASP-surgery, baseline values did not differ significantly between these groups.

**Table 1:** Data of infection parameters and heart rate.

		mild sepsis	mild sepsis	moderate sepsis	moderate sepsis
		- vasopressin	+ vasopressin	- vasopressin	+ vasopressin
IL-6 [pg/ml]	Baseline	243 ± 107	241 ± 97	459 ± 136 *	507 ± 193 *
SRSS	Baseline	3.5 ± 0.5	3.6 ± 0.8	5.5 ± 1.2 *	5.1 ± 0.7 *
HR [min^-1^]	Baseline	505 ± 49	471 ± 58	464 ± 63	476 ± 59
	30 min (6.75 mU · kg^-1^ · h^-1^)	504 ± 66	412 ± 61 #	448 ± 62	435 ± 52
	60 min (13.5 mU · kg^-1^ · h^-1^)	474 ± 74	382 ± 46 #	452 ± 54	403 ± 70 #
	90 min (27 mU · kg^-1^ · h^-1^)	468 ± 73	391 ± 44 #	437 ± 66	403 ± 68 #

Infection parameters Interleukin-6 (IL-6) and septic rat severity score (SRSS) at baseline. Heart rate (HR) [min ^-1^] of septic animals over time calculated to baseline. mild sepsis—vasopressin: Mild septic animals with vehicle-infusion; mild sepsis + vasopressin: Mild septic animals with dose escalating vasopressin-infusions; moderate septic animals with vehicle-infusion (moderate sepsis—vasopressin), moderate septic animals with dose escalating vasopressin-infusions (moderate sepsis + vasopressin). Data are presented as absolute values (means ± SD)

* = p < 0.05 versus mild sepsis (One-way ANOVA Tukey)

# = p < 0.05 versus baseline (Two-way ANOVA Dunnett); n = 10.

SRSS and IL-6 were significantly higher in moderate septic animals compared to mild septic animals, reflecting different sepsis manifestations (SRSS: 5.1 ± 0.7 (moderate sepsis + vasopressin) vs. 3.6 ± 0.8 (mild sepsis + vasopressin), p < 0.05; 5.5 ± 1.2 (moderate sepsis—vasopressin) vs. 3.5 ± 0.5 (mild sepsis—vasopressin), p < 0.05; IL-6: 507 ± 193 pg · ml^-1^ (moderate sepsis + vasopressin) vs. 241 ± 97 pg · ml^-1^ (mild sepsis + vasopressin), p < 0.05; 459 ± 136 pg · ml^-1^ (moderate sepsis—vasopressin) vs. 243 ± 107 pg · ml^-1^ (mild sepsis—vasopressin), p < 0.05 (Table 1)).

### Circulatory effect of vasopressin in moderate septic animals

Sub-therapeutic vasopressin with 6.75 mU · kg^-1^ · h^-1^ and with 13.5 mU · kg^-1^ · h^-1^ increased sublingual μHBO_2_ compared to baseline (vasopressin 6.75 mU · kg^-1^ · h^-1^: + 4.1 ± 5.5%, vasopressin 13.5 mU · kg^-1^ · h^-1^: + 5.2 ± 5.3%; p < 0.05 vs. baseline) and with 13.5 mU · kg^-1^ · h^-1^ vasopressin sublingual μHBO_2_ was significantly higher compared to moderate sepsis alone (moderate sepsis—vasopressin). In contrast, there was no difference in sublingual μHBO_2_ between therapeutic vasopressin with 27 mU · kg^-1^ · h^-1^ and between moderate sepsis alone (Fig 2A).

Moreover, sub-therapeutic vasopressin with 13.5 mU · kg^-1^ · h^-1^ increased intestinal μHBO_2_ compared to baseline (+ 6.1 ± 5.3%; p < 0.05 vs. baseline) and μHBO_2_ of the colon was significantly higher compared to moderate sepsis alone (moderate sepsis—vasopressin) (Fig 2B). Furthermore, 13.5 mU · kg^-1^ · h ^-1^ vasopressin-infusion ameliorated TVD compared to baseline (+ 5.2 ± 3.0 mm/mm^2^; p < 0.05 vs. baseline) and TVD as well as PVD were significantly increased compared to moderate sepsis alone (Fig 3B). In contrast, therapeutic vasopressin with 27 mU · kg^-1^ · h^-1^ did not change intestinal μHBO_2_ neither compared to baseline nor compared to moderate sepsis alone (Fig 2B).

Neither of the investigated vasopressin-infusions changed mean arterial blood pressure (MAP, whereas heart rate (HR) decreased significantly in all vasopressin-infusion dosages (Table 1, Fig 6B).

### Circulatory effect of moderate sepsis

In moderate septic animals (moderate sepsis—vasopressin) there were no significant changes of intestinal μHbO_2_ compared to baseline, whereas sublingual μHbO_2_ declined at 90 min (- 3.5 ± 3.8%; p < 0.05 vs. baseline) (Fig 2). TVD and PVD of the colon were not modified by moderate sepsis (Fig 3). MAP and HR remained unchanged in moderate septic animals (Table 1, Fig 6B).

### Circulatory effect of vasopressin in mild septic animals

Vasopressin-infusion with 6.75 mU · kg^-1^ · h^-1^ had no effect on sublingual and intestinal μHbO_2_, similar to vasopressin-infusion with 13.5 mU · kg^-1^ · h^-1^ or 27 mU · kg^-1^ · h^-1^ (Fig 4). 13.5 mU · kg^-1^ · h^-1^ vasopressin ameliorated TVD significantly compared to baseline (+ 4.8 ± 5.2 mm/mm^2^; p < 0.05 vs. baseline), though without changes in PVD (Fig 5). MAP did not vary due to vasopressin-infusion in the investigated dosages, whereas HR decreased significantly in all vasopressin-infusion dosages (Table 1, Fig 6A).

### Circulatory effect of mild sepsis

In mild septic animals (mild sepsis—vasopressin) sublingual as well as intestinal μHbO_2_ remained constant over time (Fig 4). TVD and PVD of the colon were not influenced by mild sepsis (Fig 5). There were also no significant changes in MAP and HR (Table 1, Fig 6A).

## Discussion

This study was carried out to evaluate the role of exogenous vasopressin with a sub-therapeutic as well as therapeutic dosing regimen on the gastrointestinal microcirculation under mild and moderate septic conditions. The main results are:

- Sub-therapeutic vasopressin ameliorates the sublingual and colonic microcirculatory oxygenation in animals with moderate but not with mild sepsis.
- The amelioration of microcirculatory oxygenation is probably mediated by a recruitment of additional capillaries.
- Exogenous vasopressin leads to a drop in heart rate whereas systemic blood pressure is not changed. However, the protective effect of vasopressin on the microcirculation is independent of changes in global hemodynamic parameters.
- Therapeutic vasopressin abolish this protective effect on gastrointestinal microcirculation during moderate sepsis. However, therapeutic vasopressin does not seem to exert detrimental effects.

To investigate the vasopressin effect in two different sepsis manifestations we used the CASP-model. This well-established model is often used to study experimental sepsis, because it closely simulates the pathophysiology of human abdominal sepsis [24]. Furthermore, by variation of stent diameter and number the CASP-model allows to induce different sepsis severities [33]. We used on the one hand a 14-gauge stent to establish a mild sepsis and on the other hand two 16-gauge stents to establish an moderate sepsis. Consecutively, the moderate sepsis group had significantly higher IL-6 levels as well as SRSS Scores indicating a more severe sepsis manifestation and is comparable to other experimental sepsis studies [9,34,35]. It is of note that an important limitation of the current study is that there is no control group to estimate how severe the sepsis-induced changes are compared to sham operated animals. However, to be in accordance with the 3 R tenet of the ARRIVE guidelines we have decided against a control group- Furthermore, as stated above, the animals showed clear signs of infection: visible peritonitis, SRSS, mortality rate (mild septic animals: 13%, moderate septic animals 20%), significantly higher IL-6 levels(and leucocyte counts compared to sham (as shown in previous studies with the same sepsis model [9,24]. Furthermore, we know from own recent studies that 24 h after CASP surgery rats do not develop septic shock. For example, animals do not suffer hypotension as expected in septic shock. Nonetheless, at the chosen time point and severity the CASP model leads to a continuously increasing systemic inflammation and provides us with the opportunity to start our therapeutic approach before the development of septic shock and to study early microcirculatory alterations of the intestine seen in the development of sepsis [24,33].

We analyzed septic microcirculation of the colon via reflectance spectrophotometry to assess alterations in the microcirculatory oxygenation. This method has been validated in various tissues and is used in different experimental and clinical studies [9,27,36–39]. Especially under septic conditions reflectance spectrophotometry is an important analyzing tool, because it measures mainly the postcapillary area and changes in this area seems to correlate with outcome in septic patients [40,41]. Furthermore, an experimental study could demonstrate that an increased intestinal oxygenation in septic rats probably leads to an improved intestinal barrier function and a reduced mucosal cell death as indirect outcome parameters, which underlines the importance of this technique [42]. Although microcirculatory oxygenation seems to be a crucial parameter, reflectance spectrophotometry alone is limited in the analysis of the microcirculation, because increased oxygenation in the postcapillary area can on the one hand be the result of increased shunting of oxygen to the venular compartment or on the other hand due to restoration of homogenous perfusion patterns, especially in sepsis [43]. Therefore, we combined the analysis of the microcirculatory oxygenation with the analysis of the microcirculatory perfusion by IDF imaging. The analysis of TVD and PVD is considered as gold standard in preclinical studies [31] and allows to differentiate the mechanisms of changes in the microcirculatory oxygenation. Furthermore, De Backer et al. have shown that changes in microcirculatory parameters recorded with IDF imaging are stronger predictors of outcome in septic patients than global hemodynamic parameters [40]. In the present study, the recorded videos were analyzed with the automatic analysis software CytoCamTools, which is recommended and similar to other analysis tools like AVA (Automated Vascular Analysis software) [32].

The chosen dosing regimen of 27 mU · kg^-1^ · h^-1^ vasopressin in our study reflects the clinically used dose of 2 U · h^-1^ vasopressin in a patient weighing 75 kg and is in line with the current guidelines of the Surviving Sepsis Campaign [17]. This dosage is considered to be therapeutic. Therefore, vasopressin dosages 6.75 mU · kg^-1^ · h^-1^ and 13.5 mU · kg^-1^ · h^-1^ are assumed to be sub-therapeutic. Vasopressin is used in intensive care medicine to restore macrohemodynamic parameters, especially blood pressure, in vasoplegic catecholamine-refractory shock states. In our sepsis study blood pressure did not change due to additional vasopressin-infusion, whereas all used vasopressin dosages led to a drop in heart rate. It is known that in rats compared to humans much higher doses of vasopressin are needed to elevate blood pressure even if the effect on heart rate is similar [19,44]. A possible explanation might be that the V1A receptors, which are essential for systemic vasoconstriction in precapillary vessels, are only 80% homologous between humans and rats [45]. However, despite these differences in the macrohemodynamic parameters, the effect of vasopressin on the intestinal microcirculation seems to be comparable between clinical and preclinical studies [18]. In therapeutically used or higher dosages of vasopressin the vasoconstrictive V1A effect seems to prevail, whereas in sub-therapeutic dosages vasodilatatory effects of vasopressin like activation of V2 receptors and oxytocin receptors appears to become more important [15,22,46,47].

These observations obtained under physiologic conditions are fully in line with the results of our study under septic conditions. Despite minor effects on macrohemodynamic parameters, we have seen profound and dose dependent changes in the intestinal microcirculation due to vasopressin. In animals with moderate sepsis sub-therapeutic vasopressin with 13.5 mU · kg^-1^ · h^-1^ improved the intestinal as well as sublingual microcirculatory oxygenation. The rise in microcirculatory oxygenation seems to be mediated by improved microcirculatory perfusion and thereby higher oxygen supply. Sub-therapeutic vasopressin-infusion with 13.5 mU · kg^-1^ · h^-1^ led to a significant increase of PVD and TVD in the colon of moderate septic animals and thereby decreased arteriovenous shunting. Hence, our study demonstrates that sub-therapeutic vasopressin ameliorated gastrointestinal microcirculation in sepsis due to recruitment of more capillaries independent of global hemodynamic parameters. This is a potential new therapeutic approach where sub-therapeutic vasopressin could be used in septic patients to restore intestinal microcirculation in sepsis.

This protective effect of vasopressin could also be demonstrated by Bomberg et al., who could show in their ischemia reperfusion model that lesser arteriovenous shunting due to vasopressin reduces hypoxic mucosal injury [48]. Our results are further supported by a study in rats of Qui et al., where low-dose terlipressin, a vasopressin analogon, improved gastrointestinal microcirculatory perfusion despite a drop in heart rate and unaltered blood pressure [49].

In contrast, our study could demonstrate that therapeutic vasopressin abolishes this protective effect but also did not show harmful effects. The gastrointestinal oxygenation did not change during vasopressin-infusion with 27 mU · kg^-1^ · h^-1^. These dose dependent effects of vasopressin are in line with a recent published trial of our study group under physiological conditions: sub-therapeutic vasopressin increased the gastric oxygenation, whereas therapeutic vasopressin did not show this amelioration [22]. This slight impact of therapeutic vasopressin on gastrointestinal microcirculation is further in line with one of the rare clinical trials. Klinzing et al. could not show any difference in hepatosplanchnic blood flow or oxygen uptake under therapeutic vasopressin-infusion in septic patients. However, gastric P_CO2_-gap increased significantly, suggesting that blood flow may have been redistributed away from the gut mucosa [21]. However, it is of note that this study has not examined if the enhanced intestinal microcirculation due to sub-therapeutic vasopressin leads to histological improvement, e.g. tight junction performance or cell death. The study focuses on microcirculatory measurements, based on recent data suggesting that even mild changes in regional oxygenation, which most likely do not lead to visible organ damage have an impact on intestinal barrier function, as shown in a canine model [50]. In this setting, i.e. in sepsis before visible tissue damage occurs, there is an opportunity for therapy in the clinical setting, whereas once the tissue is obviously damaged, interventional therapy might be too late [40]. Nevertheless, further studies have to be performed to elucidate if sub-therapeutic vasopressin can also protect organ function in sepsis.

Nevertheless, vasopressin studies should always be interpreted with care, because the overall circulatory status seems to determine the vasopressin effect in the microcirculation. Asfar et al. could demonstrate that in non-fluid-resuscitated endotoxic rats an infusion of terlipressin dramatically decreased splanchnic blood flow, whereas in fluid-challenged endotoxic rats splanchnic blood flow was well maintained [51]. Additionally, in normovolaemic endotoxic pigs even incremental doses of vasopressin infusions (14–229 mU · kg^-1^ · h^-1^) did not further compromise jejunal mucosal tissue oxygen tension and oxygen supply [52]. On the contrary, vasopressin deteriorated intestinal microcirculation in several hypodynamic shock models of sepsis, where volume status and cardiac output did not meet the enhanced needs [19,53,54]. The animals in our study did not suffer from septic shock. Instead we provided a continuous ringer infusion to establish normovolaemic conditions. Thus, our data are comparable to the clinical setting with volume therapy.

With regard to different sepsis manifestations, it is also necessary to note that a current subject of discussion is which septic patient profits from supplementary vasopressin-infusions. Especially, because a retrospective analysis of a randomized clinical trial revealed that vasopressin seems to be only beneficial in patients with less severe sepsis [23]. In the present study, we could demonstrate that in rats with moderate sepsis sub-therapeutic vasopressin seemed to be beneficial for gastrointestinal microcirculation, whereas in mild septic animals an additional vasopressin-infusion had no relevant advantageous effect. This study is one of the first studies that investigated the impact of supplementary vasopressin in different sepsis manifestations and underlines that it is of great importance to identify the appropriate patient for additional vasopressin therapy. The current sepsis guidelines recommend therapeutic vasopressin only to stabilize blood pressure in patients with septic shock. In contrast, our study could show that sub-therapeutic vasopressin could be a potentially new therapy for the restoration of intestinal microcirculation in septic patients without shock. Further research is urgently needed to reveal if sub-therapeutic vasopressin is also beneficial in hypodynamic sepsis and especially it is important to investigate sub-therapeutic vasopressin not only in experimental sepsis but also in human sepsis.

## Conclusion

Our randomized, placebo-controlled, blinded animal trial demonstrated for the first time that sub-therapeutic vasopressin improves gastrointestinal microcirculatory oxygenation in sepsis. This protective effect for the gastrointestinal mucosa seems to be mediated by enhanced microcirculatory perfusion, i.e. recruitment of additional capillaries by vasopressin, and thereby increased oxygen supply. In contrast, therapeutic vasopressin did not show this beneficial effect but also seemed to have no negative impact. Of note, we could show that is important to identify the suitable patient for additional vasopressin therapy, because sub-therapeutic vasopressin appeared to be only advantageous in rats with moderate sepsis in contrast to mild septic animals. This study reveals a potential new therapy for the compromised gastrointestinal microcirculation during sepsis and shows that clinical trials have to be performed to investigate sub-therapeutic vasopressin in septic patients.

## Supporting information

S1 Table Dataset vasopressin paper. (XLSX)
